# Influence of Friction Stir Process on the Physical, Microstructural, Corrosive, and Electrical Properties of an Al–Mg Alloy Modified with Ti–B Additives

**DOI:** 10.3390/ma15030835

**Published:** 2022-01-22

**Authors:** Essam B. Moustafa, Mashhour A. Alazwari, Waheed Sami Abushanab, Emad Ismat Ghandourah, Ahmed O. Mosleh, Haitham M. Ahmed, Mohamed A. Taha

**Affiliations:** 1 Mechanical Engineering Department, Faculty of Engineering, King Abdulaziz University, Jeddah 21589, Saudi Arabia; maalazwari@kau.edu.sa; 2 Marine Engineering Department, Faculty of Maritime Studies and Marine Engineering, King Abdulaziz University, Jeddah 21589, Saudi Arabia; wabushanab@kau.edu.sa; 3 Department of Nuclear Engineering, Faculty of Engineering, King Abdulaziz University, Jeddah 21589, Saudi Arabia; eghandourah@kau.edu.sa; 4 Mechanical Engineering Department, Shoubra Faculty of Engineering, Benha University, Cairo 11629, Egypt; ahmed.omar@feng.bu.edu.eg; 5 Department of Mining Engineering, King Abdulaziz University, Jeddah 21589, Saudi Arabia; hmahmed@kau.edu.sa; 6 Solid State Physics Department, National Research Centre, El Buhouth Street, Dokki, Giza 12622, Egypt; mtahanrc@gmail.com

**Keywords:** friction stir process, grain, refinement, Ti–B modifiers, mechanical, electrical, corrosion

## Abstract

In this study, two successive methods were used to improve the grain structure and the mechanical and physical properties of Al 5052 aluminum alloy. The modifying elements, 0.99 wt.% of titanium (Ti) and 0.2 wt.% of boron (B), were added during the casting process. After solidification, single- and double-pass friction stir processing (FSP) were performed to achieve additional grain refinement and disperse the newly formed phases well. The addition of Ti–B modifiers significantly improved the mechanical and physical properties of the Al 5052 aluminum alloy. Nevertheless, only a 3% improvement in microhardness was achieved. The ultimate strength (US), yield strength (YS), and elastic modulus were investigated. In addition, the electrical conductivity was reduced by 56% compared to the base alloys. The effects of grain refinement on thermal expansion and corrosion rate were studied; the modified alloy with Ti–B in the as-cast state showed lower dimension stability than the samples treated with the FSP method. The grain refinement significantly affected the corrosion resistance; for example, single and double FSP passes reduced the corrosion rate by 11.4 times and 19.2 times, respectively. The successive FSP passes, resulting in a non-porous structure, increased the bulk density and formed precipitates with high bulk density.

## 1. Introduction

Aluminum alloys are widely used in many industrial applications because of their lightness and malleability. The marine, aerospace, automotive, medical, and chemical industries use these alloys. Many techniques can improve the mechanical properties of aluminum alloys. Reinforcing particles and additives are essential approaches for improving the performance of these alloys [1,2,3,4]. Modification elements such as Ti, B, and B_4_C have significantly affected the grain refinement’s microstructure [5]. These modifiers are crucial for reducing the grain size and presenting a dendritic structure during the casting process, which helps in improving the characteristics of these alloys [6,7,8]. The modifiers improve the grain structure and reduce the cast alloys’ porosity; grain refinement is inextricably linked to the nucleation and development of aluminum grains; hence, this is congruent with the homogeneous and heterogeneous nucleation concepts developed by Volmer and Weber [9,10].

Inoculating particles into melts is the most effective approach to generate small, uniformly distributed, equiaxed grains, which results in great formability, high yield strength, and high toughness [11]. Over the last several decades, Al–5Ti or Al–5Ti–1B master alloys have been widely employed as aluminum grain refiners [12,13]. Many theories of grain refinement exist, with most researchers accepting the duplex nucleation hypothesis. According to duplex nucleation theory, the nuclei in crystallization are TiB_2_ particles with thin TiAl_3_ layers [14]. The TiAl_3_ intermetallic particles may function as nucleating agents for α-Al crystals [15]. Ding et al. [16] found that the arrangement of grain refinement characteristics in commercial aluminum was altered by incorporating Al–5Ti master alloys with variable sizes, morphologies, and intermetallic TiAl_3_. Pio et al. [17] showed that adding 0.5 wt% Al–5Ti–B master alloy to the alloy improved its mechanical properties. However, increasing the number of grain refiners did not provide any notable improvements.

There is another method to refine the grains of materials: in the solid state, friction stir processing (FSP) is one of the most popular and innovative technologies for refining and improving the mechanical properties of surfaces. FSP of cast A356, for example, improved the microstructure and enhanced the yield strength [18]. In addition, friction stir processing of alloy A319 improved the yield strength, ductility, and hardness [19]. Superplasticity was observed during the friction stir processing of A356 and Al7075 alloys [20,21]. FSP improved the corrosion resistance of 5083 aluminum alloy, as well as its hardness behavior due to the equiaxed recrystallized grains [22]. Many studies have explored the influence of friction stir processing (FSP) on mechanical characteristics since it enhances metals’ and composites’ mechanical and microstructural properties [23,24,25,26]. Although aluminum is corrosion-resistant, some alkali solutions attack the oxide layer on the surface and cause the surface to decay over time. Therefore, many researchers have explored corrosion inhibitors for aluminum alloys [27,28,29].

Fewer studies focus on the relationship between electrical conductivity and the mechanical properties of aluminum zinc alloys; moreover, they have revealed no such relation for other types of aluminum alloys. They do not explore the affinity of grain refinement’s influence on conductivity [30,31]. Due to reduced dislocation density in the FSPed zone and minor grain development, the thermal conductivity of the FSPed aluminum 5052 alloy rises [32]. In addition, FSP increases the electrical conductivity. For alloys, the maximum electrical conductivity was measured in the SZ centers. The electrical conductivity decreases with increasing rotation speed but remains higher than that of the base material. However, FSP decreased the corrosion resistance of the AA6063-T6 aluminum alloy. Increasing the rotation speed decreases the corrosion resistance [33].

Since thermal expansion and electrical conductivity are considered important data when entering the parameters for the modeling process into the finite element model, in addition to the material characterization process, many authors focus on these properties and their effects on the metal composition, regardless of grain refinement [24]. Based on the previous literature, the overall characterization, such as mechanical, electrical, thermal, microstructures, and corrosion behavior of the modified alloys, has not been investigated simultaneously on alloys modified with Ti–B modifiers. Furthermore, a new comparative investigation between the FSP method and modifier elements is performed here to show their effect on the mechanical, electrical, thermal, and corrosion behavior. Consequently, the current work aims to investigate the effect of using two successful processes—the use of Ti–B modifiers during the casting process and friction stir processing technology—on the various properties of the investigated alloy.

## 2. Materials and Methods

**Figure 1:**
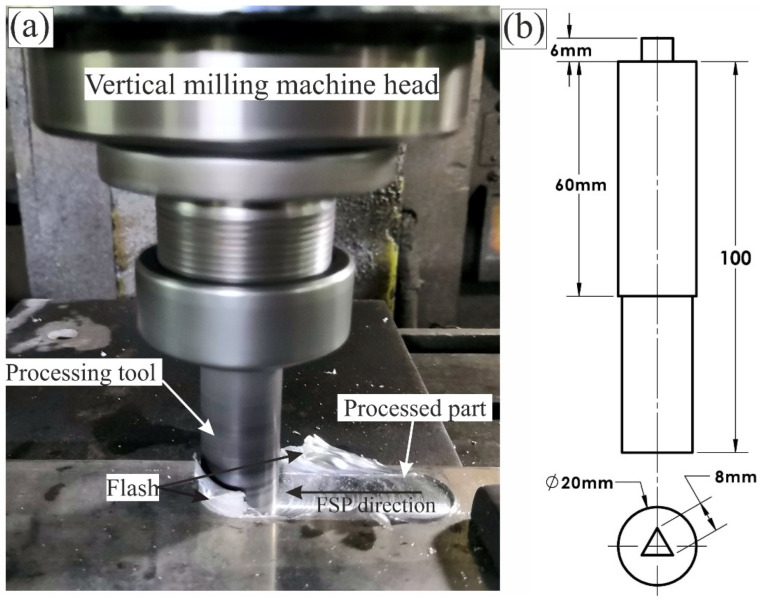
(**a**) The setup of the experimental friction stir processing and (**b**) the design of the used FSP tool.

A graphite fireclay crucible was used to melt Al 5052 and Al 5052/TiB in a resistance furnace (Nabertherm, Lilienthal, Germany). The chemical compositions of the alloys examined are summarized in Table 1. The molten components were centrifuged to mix the alloying elements with the base alloy during the casting process. An interior graphite mold measuring 250 × 70 × 15 mm^3^ was used to pour the liquid metal at 800 °C. The remaining stresses from the ingots were removed by heating in the furnace for three hours at 200 °C, followed by air cooling. An automated vertical milling machine was used to perform friction stir processing (FSP), as seen in Figure 1a (Knuth-VFM5, Knuth, Wasbek, Germany). A great deal of trial and error went into determining the machining settings. Tilt angle was set to 1, rotational speed was set at 1080 rpm, and the traverse speed was set to 30 mm per minute. Figure 1b shows the friction-stirred sample after one and two passes of treatment. The hardened K-113 tool steel (K110 Bohler steel; DIN 1.2379, AISI D2) used in the FSP tool was based on references [34,35]. An equilateral triangle with a side length of 8 mm, a shoulder diameter of 25 mm, and a pin length of 8 mm was developed and constructed for the tool. The samples were ground using a set of silicon carbide papers with grit sizes of 320, 800, 1200, 2400, and 4000 for the metallographic process. All of the samples were electrically etched using 10% electrolytes (saturated solution of H_3_BO_3_ in HF) and 90% distilled water at 18 V for 15 s. The microstructure of the specimens was studied by utilizing optical microscopy to investigate the grain microstructures (Olympus BX51, Miami, FL, USA) and scanning electron microscopy (SEM) using a Tescan-VEGA3 (model Vega 3, company TESCAN, Brno, Czech Republic, HV 20.0 kV) equipped with an X-MAX80 energy-dispersive spectroscopy (EDS) system (Oxford Instruments, Abingdon, UK). The grain size was determined using the typical linear intercept approach using the AxioVision 4.5 software attached to the microscope (Olympus BX51, Miami, FL, USA).

The XRD method “Philips PW 1373” proved the presence of the various phases in the produced samples. Following ASTM: B962-08, samples were weighed using the Archimedes technique. Using a thermal dilatometer (NETZSCH DIL 402 PC, Selb, Germany) with a heating rate of 5 °C/min and rectangular bars, we measured the samples’ thermal expansion from 25 to 500 °C, as detailed in our previous work [5]. The samples were tested for compressive strength using the ASTM E9–19 standard. As previously mentioned [36,37], the speed of longitudinal and shear waves at room temperature was measured using the pulse-echo method using an ultrasonic digital signal processing system (Model MBS8000 DSP, MATEC Instrument Companies, Northborough, USA) with 5 MHz resonance. Static immersion weight loss was used at ambient temperature (25 °C) to assess the corrosion rate of sintered samples. Diamond paste was used to provide a mirror-like finish on the samples’ surfaces after being polished with SiC papers of different grit levels up to 2000 grits. Additionally, each sample was measured and placed into a 3.5 wt.% NaCl solution before being extracted after 1 to 8 days in the solution. Samples were also tested for electrical conductivity using a Keithley 6517B system (6517 B, Keithley Instruments, OH, USA) at ambient temperature (26 °C) and 40 V.

## 3. Results and Discussion

### 3.1. Microstructure Analysis

**Figure 2:**
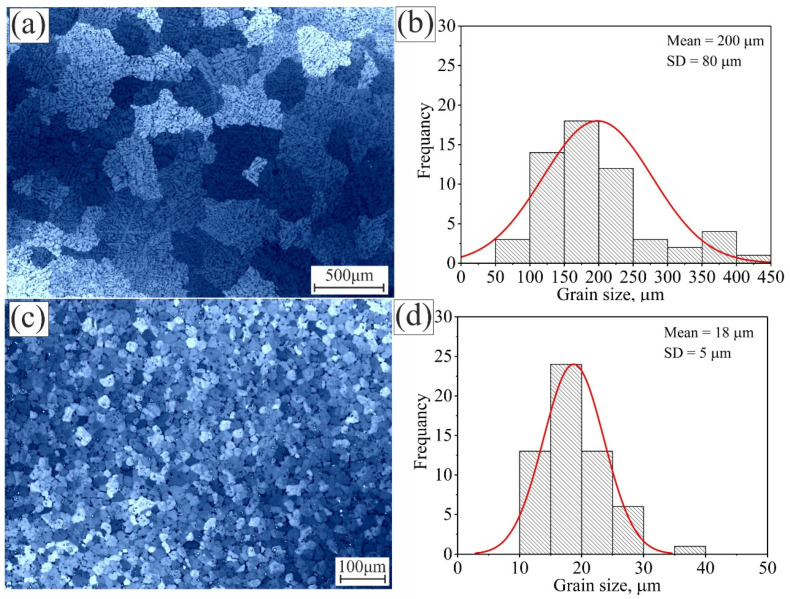
The as-cast microstructure and grain size distribution for (**a**,**b**) Al 5052 and (**c**,**d**) Al 5052/TiB.

Figure 2 shows the microstructure and grain size distribution of the Al 5052 and Al 5052 alloys after casting. Al 5052 had large grains, with a coarse dendritic structure (Figure 2a). The average grain size of Al 5052 after casting was 200 ± 15 µm. The grain size was broadly distributed around the mean, with a standard deviation (SD) of 80 µm (Figure 2b). The inclusion of the Ti–B modifier during the casting process refined the grains and eliminated the dendritic structure (Figure 2c). The average grain size of Al 5052/TiB after casting was 18 ± 3 µm. The grain size was closely distributed around the mean, with a standard deviation of 5 µm (Figure 2d). 

**Figure 3:**
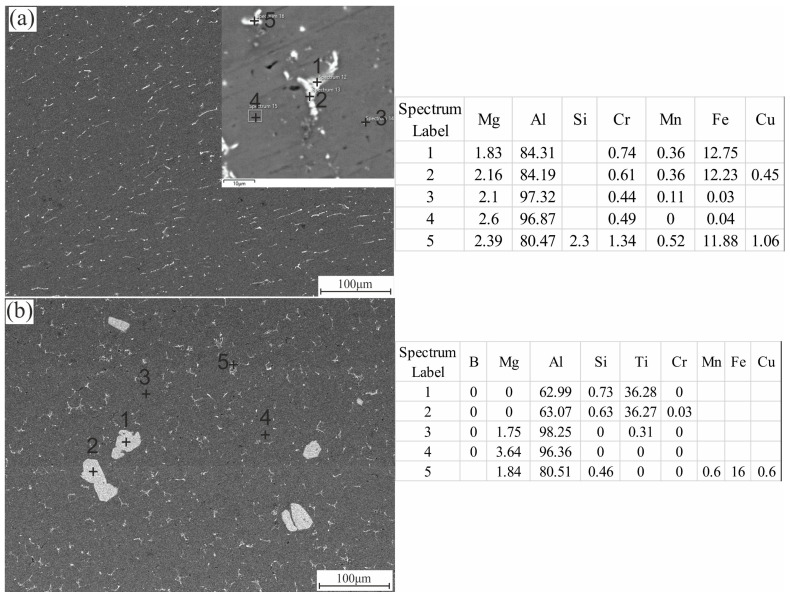
The as-cast SEM and EDS analysis of (**a**) Al 5052 and (**b**) Al 5052/TiB alloys (all values are in wt%).

Figure 3 shows the SEM micrographs and EDS point analysis of the investigated alloys in the as-cast state: Al 5052 (Figure 3a) and Al 5052/TiB (Figure 3b). It can be seen that the usual intermetallic phases, Mg_2_Si and Al_3_Fe, were formed between the dendrite arms of the standard Al 5052 alloy (Figure 3a). The distribution of the interdendritic phases confirms the uniformity and regularity of the grains. The other elements were dissolved in the Al-alpha solid solution (Figure 3a, points 3 and 4). Modifying the Al 5052 alloy with Ti–B resulted in the formation of phases containing Ti and B, such as Al_3_Ti and TiB, which acted as nucleation centers during solidification and led to fine grains (Figure 3b). The XRD analysis also confirmed the formed phases, as described in the next section.

**Figure 4:**
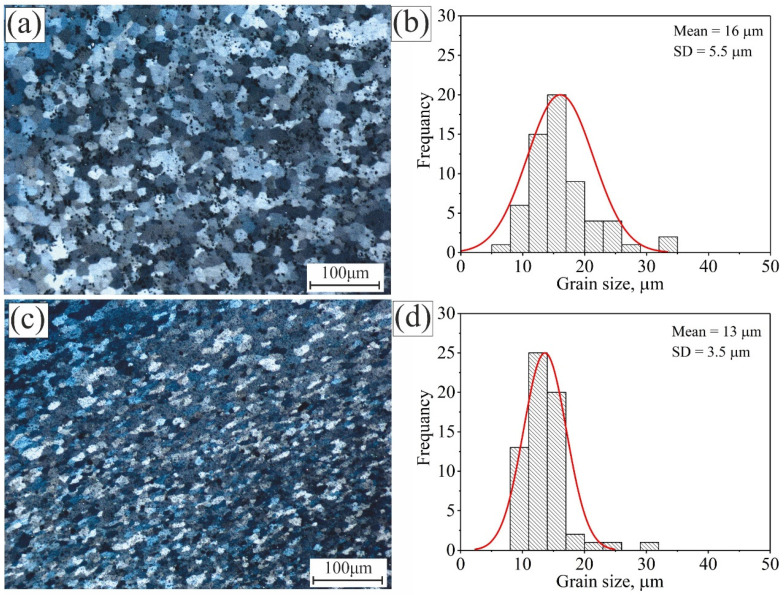
The microstructure and the grain size distribution in the SZ after the first pass of the investigated alloys: (**a**,**b**) Al 5052 and (**c**,**d**) Al 5052/TiB.

Figure 4 shows the micrographs and grain size frequency of the standard alloy Al 5052 and the modified alloy with 0.99%Ti + 0.2%B after the first pass of the friction stirring process in the stirred zone (SZ). After the first pass of FSP on Al 5052, the grains became homogeneous and equiaxed, and the dendritic structure disappeared from the SZ (Figure 4a). The average grain size in the SZ decreased to 16 ± 3 µm, and the grains were normally distributed around the mean, with SD = 5.5 µm (Figure 4b). The grains were refined by 12.5 folds, showing the soundness of FSP in modifying the microstructure of the as-cast dendritic structure alloys. The modified Al 5052/TiB alloy grains were insignificantly refined after FSP, and the average grain size decreased to 13 ± 2 µm. The grains were distributed around the mean value, with SD = 3.5 µm.

**Figure 5:**
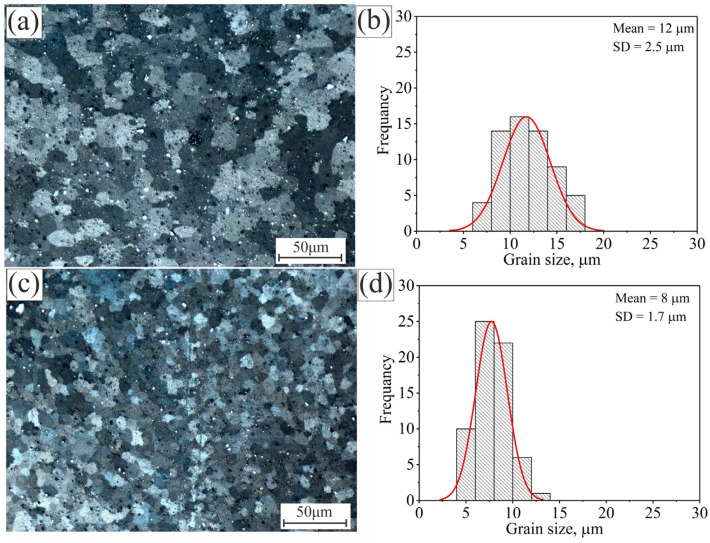
The microstructure and the grain size distribution after second pass of the investigated alloys: (**a**,**b**) Al 5052 and (**c**,**d**) Al 5052/TiB.

Figure 5 shows the micrographs and grain size abundance of the standard Al 5052 alloy and the modified alloy with 0.99%Ti + 0.2%B after the second pass of the friction stir process in the SZ. In general, the second pass of FSP resulted in greater refinement of the grains (Figure 5a,c). The average grains of the Al 5052 alloy and the modified Al 5052/TiB alloy were reduced to 12 ± 2 and 8 ± 2 µm, respectively. The grains of both alloys were normally distributed around the mean after the second pass, with SD of 2.5 and 1.7 µm, respectively (Figure 5b,d). After two FSP passes, the total grain refinement was 17 and 2.5 times, respectively, compared to the base alloys. The incremental refinement process for Al 5052/TiB was approximately 25 times; the first step in casting was 11-fold, and the second step after two FSP passes was 2.5-fold; therefore, the incremental refinement process for this alloy was approximately 25-fold.

### 3.2. Hardness Behavior

**Figure 6:**
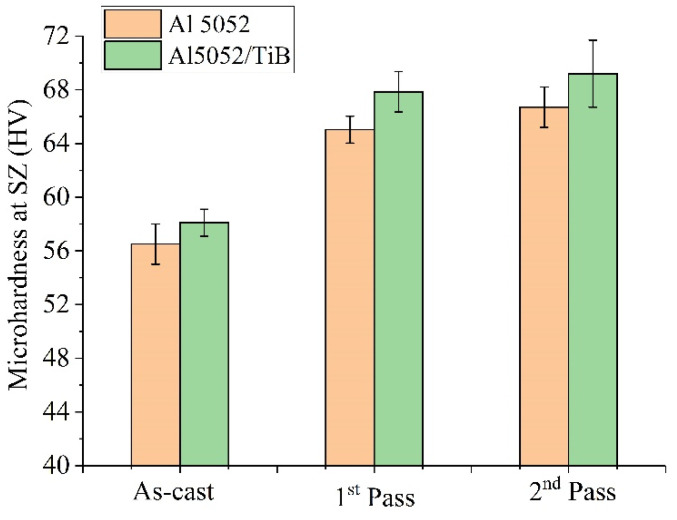
Microhardness of the investigated alloys in the as-cast state and FSP after first- and second-pass friction stir processing.

Hardness is often used as a reference for other mechanical characteristics. Vickers hardness values for the standard alloy Al 5052 and the modified alloy with 0.99%Ti + 0.2%B in the as-cast condition and FSP after the first- and second-pass friction stir processing are shown in Figure 6. It can be observed that the modification of the Al 5052 alloy with Ti–B increased the hardness after casting. The increased hardness resulted from grain refinement and the formation of Al_3_Ti and TiB_2_. In general, the FSP showed a positive effect on the SZ hardness for the studied alloys. For Al 5052, the hardness increased from 56 ± 2 HV to 65 ± 2 HV after the first pass. After the second pass, further improvements in hardness were recorded. Al 5052/TiB was also characterized in the same way.

### 3.3. XRD Analysis

**Figure 7:**
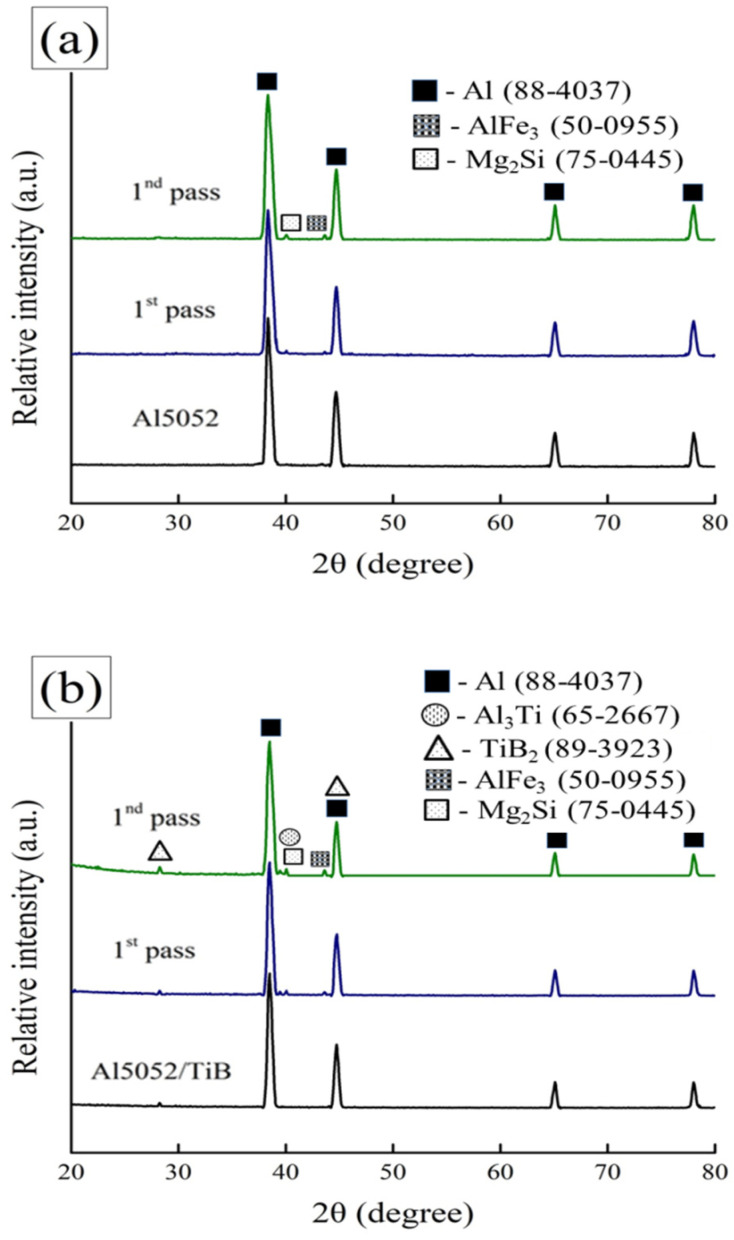
XRD patterns of single and double pass of the investigated samples: (**a**) Al 5052 and (**b**) Al 5052/TiB.

Figure 7a,b show the results of the XRD analysis of Al 5052 and Al 5052/TiB and the single and double passes of FSP. It can be seen from Figure 7a that only Al peaks appeared in the as-cast state. After a single pass of FSP, a weak peak appeared for Mg_2_Si and another for Al_3_Fe, and after the double-pass FSP, Mg_2_Si and Al_3_Fe peaks appeared more clearly. For Al 5052/TiB in the as-cast state (Figure 7b), the Al peaks appeared in addition to a small and weak TiB_2_ peak, while, for Al 5052, after one pass of FSP, the same peaks appeared in addition to the weak Al_3_Ti peak; moreover, for double-pass FSP, the TiB_2_ and Al_3_Ti peaks appeared clearly. After the addition of Ti–B, the Al_3_Ti phase was formed as a result of the reaction of the Al base with the Ti additive during the casting process and increased during the FSP process. The formation of these phases is important in influencing various properties, such as the mechanical properties, coefficient of thermal expansion, and corrosion behavior, which will be discussed in the following sections.

### 3.4. Bulk Density

**Figure 8:**
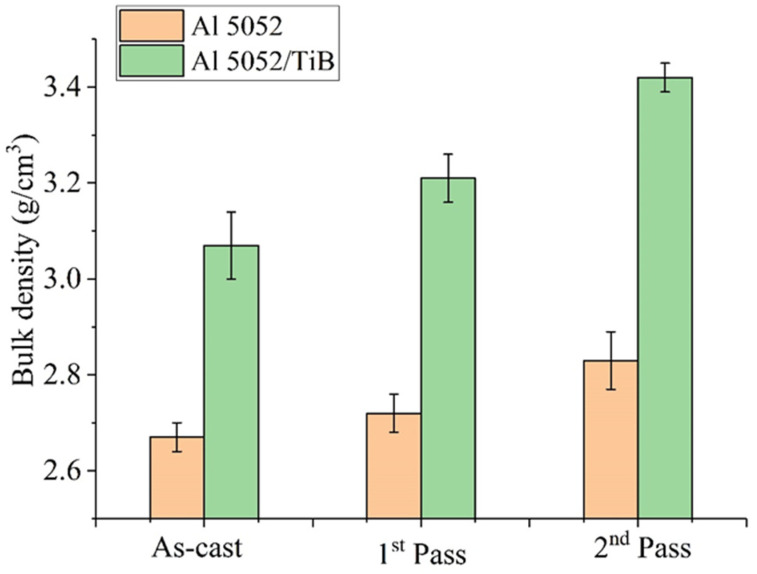
Bulk density values of the samples.

Figure 8 shows the effects of Ti–B particles on the bulk density of the Al 5052 alloy before and after FSP. As can be seen, the density of the Al alloy increased not only due to the addition of the Ti–B content but also due to the increase in the passes of the FSP. Adding Ti–B leads to the refinement of the grains without forming any cavitation, resulting in higher bulk density compared to the Al 5052 alloy. The increase in the bulk density after the first- and second-pass friction stir processing can be better explained by the absence of voids and stir casting defects. On the one hand, FSP is responsible for a significant increase in the temperature at the surface contacts between the particles, which is associated with the formation of closed pores and grain growth. On the other hand, the bulk density of TiB_2_ = 4.53 g/cm^3^ is almost twice that of the Al alloy = 2.68 g/cm^3^. The sequential single- and double-pass FSP, resulting in a pore-free structure, led to an increase in the bulk density, in addition to forming high-bulk-density precipitates.

## 4. Thermal Expansion

**Figure 9:**
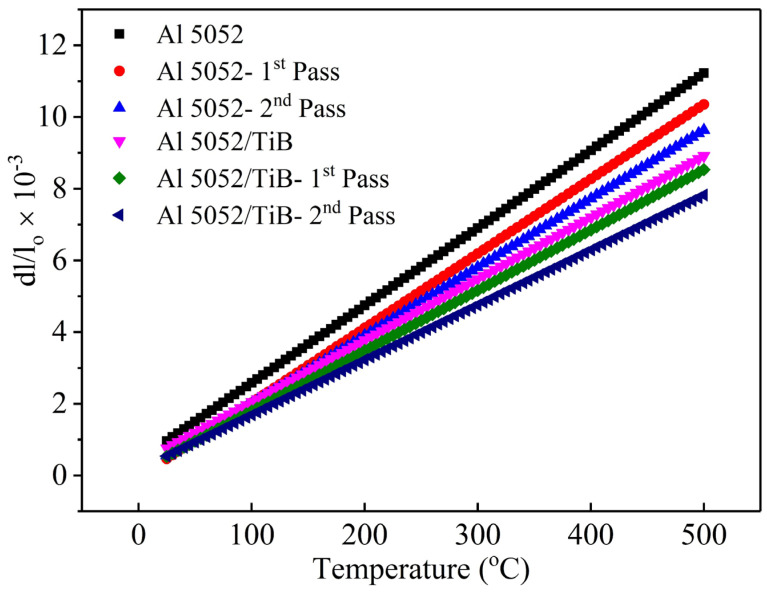
Thermal expansion behavior of the samples.

The relative thermal expansion (∆L/L) of the Al 5052 and Al 5052/TiB alloys before and after a single and double pass of FSP, measured at temperatures between 25 and 500 °C, is shown in Figure 9. As expected, the results illustrate that the relative thermal expansion of Al 5052 and Al 5052/TiB alloys increased with increasing temperature, considering that their values were not strongly affected by the single and double passes of FSP compared to the samples before FSP treatment. It can be observed that the addition of Ti–B had a significant effect on the relative thermal expansion since it caused a decrease in the ∆L/L value, which was more pronounced with increasing temperature. This can contribute to the grain refinement after casting and highly thermally stable elements, Ti and B, compared with the other elements. For alloy Al 5052, measured at 200 °C, the ΔL/L value was 4.75 × 10^−3^. After single and double passes, the ∆L/L values increased to 4.12 × 10^−3^ and 3.91 × 10^−3^, respectively. If the temperature was increased to 500 °C for the same sample, the ΔL/L value was 11.22 × 10^−3^, and after single and double passes, it was 10.36 × 10^−3^ and 9.64 × 10^−3^, respectively. For the Al 5052/TiB sample measured at 200 °C, the ΔL/L value was 3.77 × 10^−3^, and after one and two passes, it was 3.49 × 10^−3^ and 3.23 × 10^−3^, respectively. On the other hand, if the measurement temperature for the same sample was increased to 500 °C, the ΔL/L value changed to 8.91 × 10^−3^, and after one and two passes, it was 8.53 × 10^−3^ and 7.83 × 10^−3^, respectively.

**Figure 10:**
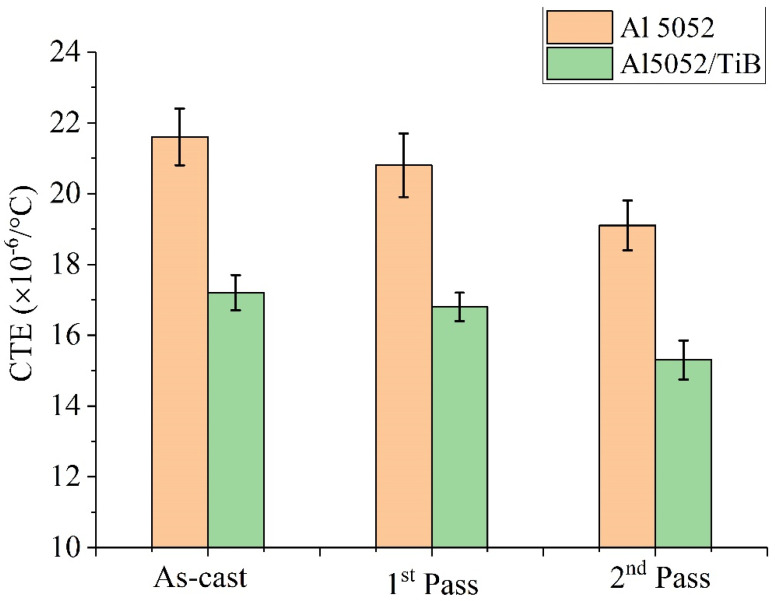
The effect of FSP and added Ti–B on the CTE values of Al5052.

The measured coefficient of thermal expansion (CTE) values for the same samples are shown in Figure 10. The CTE value showed the same trend for ΔL/L, where there was a slight decrease after FSP for the base alloy, while it decreased significantly with the addition of Ti–B (Al 5052/TiB samples). The CTE value for Al 5052 and Al 5052/TiB was 21.6 and 17.2, respectively. After one pass, the values were 20.8 and 16.8, respectively. However, after two passes, the values were 19.1 and 15.3, respectively. The results indicate that the CTE values showed the same trend of thermal expansion as the CTE value of the base alloy, which slightly decreased after FSP due to the formation of the Mg_2_Si and Al_3_Fe phases, which are characterized by a lower CTE compared to the Al 5052 alloy.

On the other hand, for the Al 5052/TiB sample, the significant decrease in the CTE value occurred due to the formation of the highly thermally stable phase TiB_2_ (CTE ≈ 7.1 × 10^−6^/°C) [38]. Moreover, residual stresses caused by thermal mismatch between the Al 5052 alloy and TiB_2_ phase play an important role in determining the thermal expansion behavior of alloys [39]. Thus, the thermal stability in the dimensions of aluminum alloys can be increased by adding Ti–B elements and using FSP. The Ti–B elements have low thermal expansion, and the FSP forms highly stable phases.

## 5. Mechanical Properties

**Figure 11:**
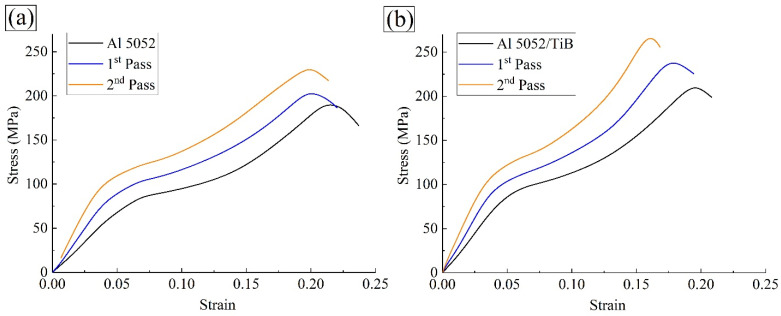
Stress–strain curve of (**a**) Al 5052 and (**b**) Al 5052/ TiB for one and two passes of FSP.

**Figure 12:**
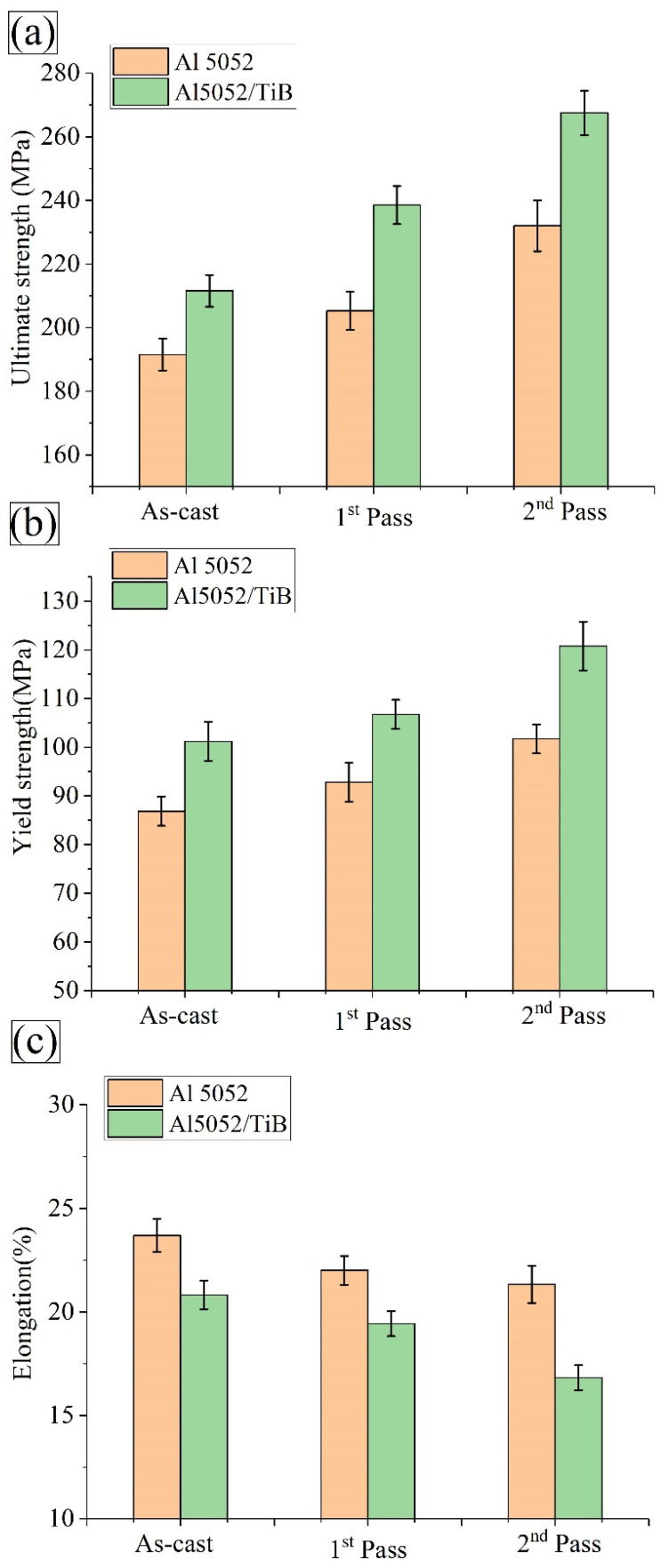
(**a**) Ultimate strength, (**b**) yield strength, and (**c**) elongation of Al 5052 and Al 5052/TiB samples for one and two passes of FSP.

Figure 11a,b show the stress–strain curves of the Al 5052 and Al 5052/TiB alloys for one and two passes of FSP. Moreover, based on the compressive yield curves in Figure 12, the main compressive properties such as yield strength (YS), ultimate strength (US), and elongation (E), were determined for the prepared specimens at different FSP passes (see Figure 12a–c). This figure shows that the alloy Al 5052 exhibited values of 86.8 MPa, 191.5 MPa, and 23.7% for YS, US, and E, respectively. After the FSP process, both YS and US were improved and increased to 92.8 and 205.2 MPa, respectively, after one pass, while E decreased by 22%. After two passes, the results show a further significant improvement in the strength, with values of 101.7 and 232, respectively. Surprisingly, these passes led to a 21.3% decrease in strain. In fact, the TiB_2_ phase was very hard in the Al 5052/TiB alloy and greatly impacted the compressive properties, except for the strain. The ultimate strength of the Al 5052/TiB alloy, after the first pass and the second pass, amounted to 211.6, 238.6, and 267.6 MPa, respectively, which are equal to 10.5, 16.3, and 15.3% increases compared to the Al 5052 alloy, while the elongation for the same specimens amounted to 20.8, 19.4, and 16.8%, respectively.

**Figure 13:**
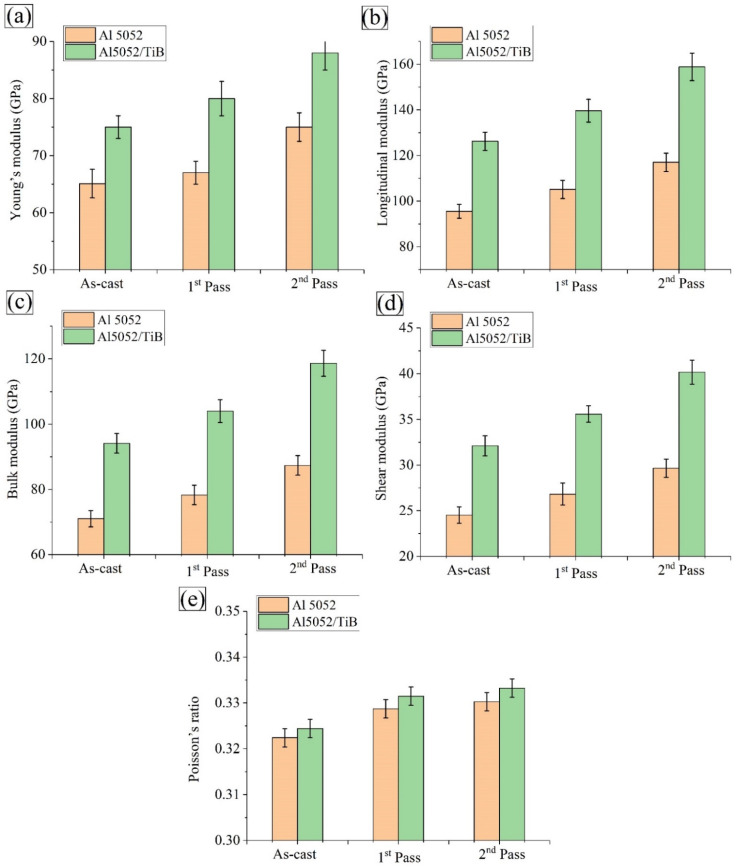
Elastic moduli of Al 5052 and Al 5052/TiB samples: (**a**) young’s modulus, (**b**) longitudinal modulus, (**c**) bulk modulus, (**d**) shear modulus and (**e**) Poisson’s ratio.

Figure 13 shows the effects of the FSP passes and the addition of Ti–B on the elastic moduli (i.e., E Figure 13a, L Figure 13b, K Figure 13c, S Figure 13d, and ν Figure 13e) for Al 5052. It can be seen that the values of the elastic modulus experienced the same trend as the yield strength and ultimate strength when both Ti–B and FSP were added. For example, the longitudinal modulus of the Al 5052 alloy was 95.5 GPa, and after FSP was added twice, the value was 117, which is equal to a 22% increase. With the addition of Ti–B, the value of the elastic modulus increased to 75 GPa, which corresponds to an increase of approximately 16% compared to the Al alloy. After FSP, the value was 88 GPa, which corresponds to an increase of approximately 35.7% compared to the Al alloy after FSP.

The apparent improvement in the mechanical properties of the Al 5052 alloy was represented by the US, YS, and elastic moduli after the FSP process. Interestingly, this improvement can be attributed to grain fragmentation, work hardening, and precipitates’ partial formation. Thus, with the increase in grain boundaries and recrystallized grains in the SZ of Al 5052 and Al 5052/TiB, the maximum mechanical properties were observed in one and two passes, respectively [40,41]. On the other hand, the addition of reinforcing agents from the ceramic phase (TiB) within Al 5052 by the FSP process improved the final US and YS according to the strengthening mechanisms. Nevertheless, a decrease followed it in L, which is consistent with much of the literature dealing with the improvement of the mechanical properties of Al alloys by the addition of different ceramics [4,5,42]. Moreover, the homogeneous distribution of TiB_2_ particles in the Al alloy may obstruct the dislocation motion. As a result, dislocation loops are formed around the TiB_2_ particles, which increase the stress required for further deformation (Orowan strengthening mechanism) [43].

## 6. Corrosion Behavior

**Figure 14:**
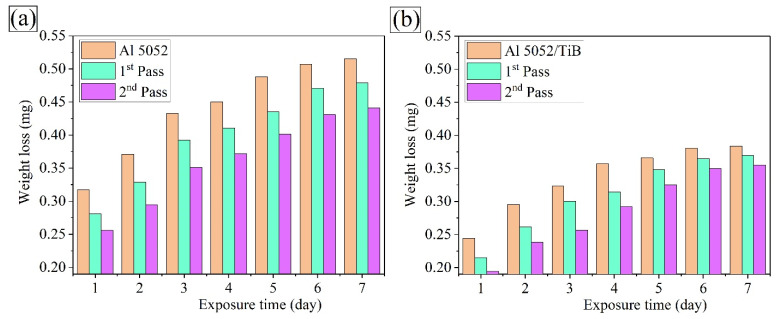
Weight loss of the investigated samples (**a**) Al 5052 and (**b**) Al 5052/TiB.

**Figure 15:**
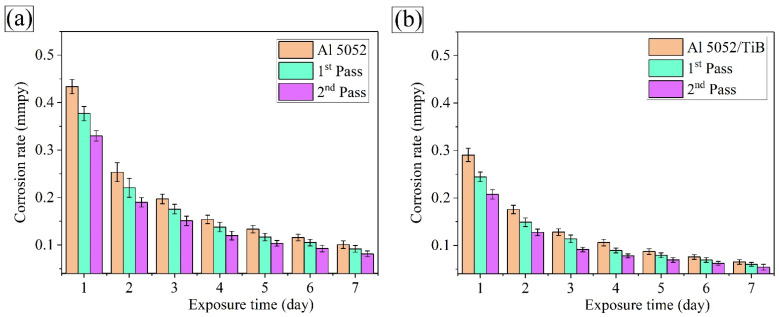
Corrosion rate of the investigated samples (**a**) Al 5052 and (**b**) Al 5052/TiB.

The weight loss approach was considered better than other approaches to evaluate metal corrosion in an immersion test. Figure 14 and Figure 15 show the weight loss variation and corrosion rate of Al 5052 and Al 5052/TiB before and after FSP with an exposure time of 3.5 wt.% NaCl solution at room temperature. In this context, the weight loss measurements were studied at intervals of 1, 2, 3, 4, 5, 6, and 7 days. The weight loss of the Al 5052 sample immersed for 1, 4, and 7 days was 0.317, 0.450, and 0.515 mg, respectively; after the single pass at the same immersion times, values were 0.281, 0.411, and 0.479 mg, respectively, and after the double pass, they were 0.256, 0.372 and 0.441 mg, respectively. On the other hand, for the Al 5052/TiB sample, the weight loss for the same immersion time was 0.244, 0.357, and 0.384 mg, respectively; after the single pass, values were 0.28115, 0.314, and 0.370 mg, respectively, and after the double pass, they were 0.194, 0.292 and 0.355 mg, respectively. Figure 14 shows the change in the corrosion rate of Al 5052 and Al 5052/TiB before and after FSP at the above exposure times. It can be seen that the corrosion rate of Al 5052 alloy after one day of immersion was 0.317 mmpy (Figure 15a). In the case of single and double immersion, the corresponding corrosion rates were 0.281 and 0.256 mmpy, respectively. This shows that the corrosion rate was reduced by 11.4 times in the case of a single pass and 19.2 times in the case of a double pass compared to the corrosion rate of the starting alloy. Al 5052/TiB was found to have a corrosion rate of 0.244 mmpy (Figure 15b), an increase of ~123% over Al 5052. After single and double passes, the corrosion rate was 0.215 and 0.194 mmpy, respectively, which corresponds to a reduction of ~32.2% for a single pass and 38.8% for double passes compared to the corrosion rate of the Al 5052 alloy. Since the corrosion process generally takes place on the surfaces of samples, it is interesting to note that the weight loss increased at the beginning of the test. As a result of passivation, it tended to remain stable at the end of the test.

Consequently, an increase in the exposure time led to a decrease in the corrosion rate. Obviously, after a single pass, the possibility of corrosion decreases because significant grain boundaries have formed during FSP due to grain refinement. However, the passive surface film consists of high-density grain boundaries in refined grain regions, resulting in a lower corrosion rate [44]. Moreover, the formation of the perceptual phase (Mg_2_Si and Al_3_Fe) during FSP is expected to contribute to the decrease in the CTE values, thus improving the thermal stability of the samples. There were significant improvements in the CTE value after adding Ti–B reinforcement to the Al 5052 alloy due to the formation of the TiB_2_ ceramic phase, where the ceramic particles remained intact, i.e., with no discernible corrosion behavior, and the samples protect the surface layer in an acidic medium [39,45].

## 7. Electrical Conductivity

**Figure 16:**
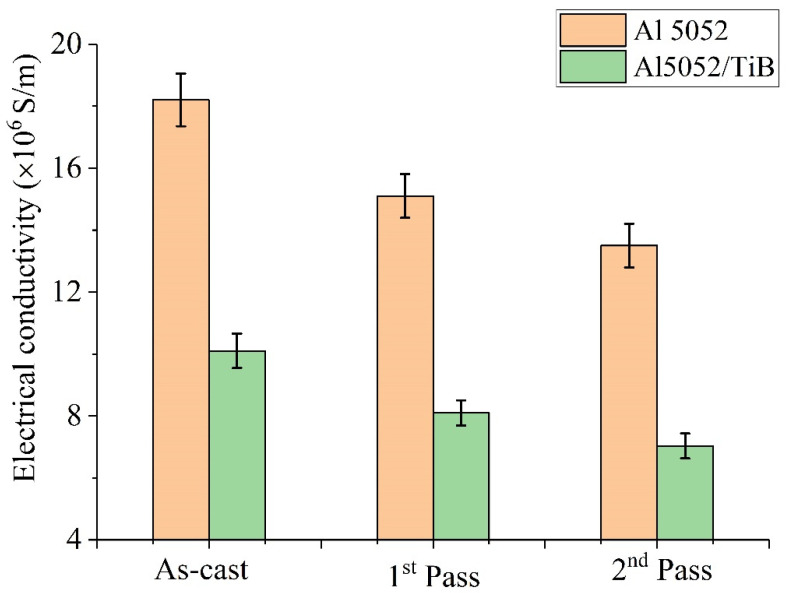
Electrical conductivity of Al 5052 and Al 5052/TiB samples.

The electrical conductivity of the samples before and after FSP is shown in Figure 16. The results show that the electrical conductivity of the Al 5052 alloy in the as-cast state was 1.82 × 10^7^ S/m and slightly decreased after FSP, reaching 1.51 × 10^7^ and 1.35 × 10^7^ S/m for the single- and double-pass FSP, respectively. This decrease in conductivity value could be related to the increase in the number of grain boundaries caused by the fine-grained Al alloy due to FSP, which hinders the electron flow and thus decreases the conductivity. Another possible reason is the formation of precipitate phases, i.e., Mg_2_Si and Al_3_Fe (as seen in the X-ray image), which reduce the conductivity due to the lack of electrical conductivity of the Al alloy. On the other hand, the conductivity of the Al 5052/TiB sample decreased by 20% to 1.01 × 10^7^ S/m compared to the Al alloy. After one and two passes, the conductivity decreased by 20 and 30% to 8.10 × 10^6^ and 7.08 × 10^6^ S/m, respectively, compared to the Al alloy after FSP. This decrease in conductivity due to the presence of ceramic TiB_2_ leads to an increase in the number of electron-scattering interfaces between the Al alloy and TiB_2_ phase. Moreover, the presence of this phase increases the number of pores that act as barriers to electron flow, thus decreasing the electrical conductivity.

## 8. Conclusions

In the current work, the Al 5052 alloy was modified using two successive methods: Ti–B modification and surface refinement using the FSP technique. The study showed that the surface is finer and has better mechanical and physical properties. In addition, the resulting surface was studied using the sequential method after each of the above refinement methods.

Al 5052 alloy contains large grains, with a coarse dendritic structure and an average grain size of 200 ± 15 µm. The addition of Ti–B to the Al 5052 alloy resulted in creating Al_3_Ti and TiB, which serve as nucleation nuclei during solidification. After the first FSP pass, the grains in Al 5052 were homogeneous and equiaxed, and the dendritic structure had vanished in the stirred zone (SZ). After the second FSP pass, the overall grain refinement was 17 and 2.5 times that of the basic alloys.

After stir casting, the addition of Ti–B to the Al 5052 alloy enhanced the hardness. The rise in hardness was caused by grain refinement and the production of Al_3_Ti and Ti–B. In general, the FSP enhanced the SZ hardness of the alloys examined.

The FSP enhanced the mechanical characteristics of the Al alloys following grain refinement by raising the US, YS, and Young’s moduli. When Al 5052/TiB was exposed to double FPS passes, the mechanical characteristics were improved.

The successive first and second FSP passes, leading to a non-porous structure, caused an increase in bulk density and the formation of high-bulk-density precipitates. In addition, the inclusion of Ti–B constituents and the use of FSP can improve the thermal stability in aluminum alloy dimensions. Ti–B elements have low thermal expansion and FSP leads to the formation of very stable phases.

The electrical conductivity of the Al 5052 alloy was 1.82 × 10^7^ S/m, which was reduced somewhat after FSP. The increase in the number of grain boundaries induced by the fine-grained Al alloy might account for the drop in conductivity. Another factor might be the production of precipitate phases, such as Mg_2_Si and Al3Fe, which reduce conductivity.

With one pass, corrosion was decreased by 11.4 times, and with two passes, the corrosion rate was reduced by a factor of almost 20.

## Figures and Tables

**Table 1:** The chemical composition of the standard Al 5052 alloy and that modified with Ti–B (wt.%).

Alloy	Si	Cu	Fe	Mn	Mg	Zn	Cr	Ti	B	Al
Al 5052	0.55	0.11	0.39	0.10	2.57	0.15	0.3	-	-	remainder
Al 5052 + Ti–B	0.71	0.12	0.38	0.11	2.54	0.13	0.23	0.99	0.2	remainder

## Data Availability

The data presented in this study are available on request from the corresponding author.
